# A Review of Ten Years of the Study for Monitoring Antimicrobial Resistance Trends (SMART) from 2002 to 2011

**DOI:** 10.3390/ph6111335

**Published:** 2013-11-01

**Authors:** Ian Morrissey, Meredith Hackel, Robert Badal, Sam Bouchillon, Stephen Hawser, Douglas Biedenbach

**Affiliations:** 1 IHMA Europe Sàrl, 9A Route de la Corniche, Epalinges 1066, Switzerland; 2 International Health Management Associates, Inc; Schaumburg, IL 60173, IL, USA

**Keywords:** carbapenems, surveillance, Gram-negative, extended-spectrum β-lactamase

## Abstract

Surveillance of antimicrobial agent resistance provides important information to guide microbiologists and infectious disease specialists understanding of the control and the spread of resistance mechanisms within the local environment. Continued monitoring of antimicrobial resistance patterns in the community and in local hospital environments is essential to guide effective empiric therapy. The Study for Monitoring Antimicrobial Resistance Trends (SMART) has monitored the *in vitro* susceptibility patterns of clinical Gram-negative bacilli to antimicrobial agents collected worldwide from intra-abdominal infections since 2002 and urinary tract infections since 2009. Resistance trends, with a particular focus on carbapenem resistance and the rate of extended-spectrum β-lactamases (ESBLs), were analyzed. Isolates from intra-abdominal infections (n = 92,086) and urinary-tract infections (n = 24,705) were collected and tested using Clinical and Laboratory Standards Institute methods. This review presents carbapenem susceptibility and ESBL rates over ten years of SMART study analysis, including key publications during this period. The SMART study has proved to be a valuable resource in determining pathogen prevalence and antibiotic susceptibility over the last ten years and continues to provide evidence for regulatory susceptibility breakpoints and clinical decision making.

## 1. Introduction

Surveillance of antimicrobial resistance in frequently isolated human pathogens can help to develop rational therapies [1]. Hospital studies can guide local antimicrobial agent use or larger studies can collect isolates for resistance testing at a central microbiology laboratory. Data collected from such studies can be used to guide microbiologists and infection disease specialists in the control and containment of resistant pathogens. Although antibiotics have been in use since the 1940s, coordinated multicentre surveillance studies were not considered until the early 1990s. One of the earliest of these surveillance studies was the Alexander project which began in 1992 and focused on respiratory tract infections [2]. Since then, other global studies have provided additional scientific data with regards to antimicrobial resistance. These include studies such as EARS-net, PROTEKT, SENTRY and TEST which are discussed elsewhere [1].

The Study for Monitoring Antimicrobial Resistance Trends (SMART) is an ongoing global surveillance study. This investigation has determined the *in vitro* resistance of clinical Gram-negative bacilli to antimicrobial agents from intra-abdominal infections (IAIs) since 2002 and was extended further to provide antimicrobial resistance data and pathogen prevalence from urinary tract infections (UTIs) in 2009 worldwide, with a particular focus on carbapenems and extended-spectrum β-lactamases (ESBLs). The two main objectives of the SMART study were to monitor the *in vitro* resistance in Gram-negative bacilli to antimicrobials IAIs and UTIs and identify changes in resistance patterns in community- or hospital-acquired organisms, including those that produce ESBLs. This review presents some newly analysed data for carbapenem resistance and ESBLs over ten years of the SMART study and discusses key publications from the study during this period.

## 2. Materials and Methods

### 2.1. Isolate Collection and Sites

**Figure 1:**
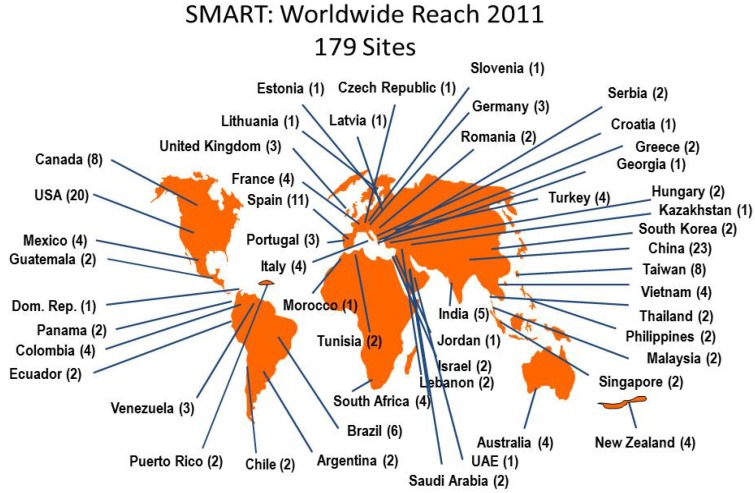
Map indicating SMART sites as participating in 2011.

Hospital laboratories from multinational locations were invited to collect up to 100 consecutive Gram-negative bacilli from patients with intra-abdominal infections and 50 Gram-negative isolates from urinary-tract infections each year. The collection only includes unique bacterial isolates and excludes duplicate isolates. Isolates were differentiated on the duration of hospitalization (<48 h or ≥48 h) at time of isolate recovery. Isolates recovered at <48 h of hospitalization were considered community-associated and those recovered at ≥48 h of hospitalization were considered to be hospital-associated. Isolates were identified and confirmed as Gram-negative bacilli and then shipped to a central microbiology laboratory (International Health Management Associates, Inc, Schaumburg, IL, USA). These isolates were re-identified at the central laboratory using biochemical methods as appropriate. In total 282 hospital sites have participated in the SMART program since 2002. The number of participating sites has grown from 40 in 2002 to 179 in 2011. The location of these laboratories for 2011 is shown in Figure 1. There were 165 sites that participated in three or more years; 71 that participated for five or more years; and 13 sites have participated in all 10 years.

### 2.2. Susceptibility Testing

Isolates were tested for antimicrobial susceptibility using custom MicroScan dehydrated broth microdilution panels (Siemens Medical Solutions Diagnostics, West Sacramento, CA, USA) at the central laboratory. Minimum inhibitory concentrations (MICs), Clinical and Laboratory Standards Institute (CLSI) guidelines were utilized for testing [3]. The following antimicrobial agents were included on the panels with their dilution ranges (expressed in mg/L):

- ertapenem 0.03–4,
- imipenem 0.06–8,
- cefepime 0.5–32,
- ceftazidime 0.5–128,
- ceftazidime-clavulanic acid 0.12/4–16/4,
- cefoxitin 2–16,
- ciprofloxacin 0.25–2,
- amikacin 4–32,
- levofloxacin 0.5–4,
- cefotaxime 0.5–128,
- cefotaxime-clavulanic acid 0.12/4–16/4,
- piperacillin-tazobactam 2/4–64/4,
- ampicillin-sulbactam 2/2–16/2, and
- ceftriaxone 1–32.

MIC interpretive criteria of the most recent CLSI documents were followed for all pathogens and antimicrobial agents [4].

Using CLSI guidelines, *Eschericia coli*, *Klebsiella pneumoniae*, *K. oxytoca*, and *Proteus mirabilis* were classified as ESBL producers if there was at least an eight-fold reduction (*i.e.*, three doubling dilutions) of the MIC for ceftazidime or cefotaxime tested in combination with clavulanic acid *versus* their MIC values when tested alone [4]. Trends were assessed for statistical significance using the Cochrane-Armitage test.

### 2.3. Quality Control

Quality control testing (QC) was performed each day of testing using the CLSI recommended American Type Culture Collection (ATCC) QC strains: *E. coli* ATCC 25922, *E. coli* ATCC 35218, *Pseudomonas aeruginosa* ATCC 27853, and *K. pneumoniae* ATCC 700603 (positive ESBL control). Results were included in the analysis only when corresponding QC isolates tested within the acceptable range according to CLSI guidelines [4].

## 3. Results and Discussion

Over the course of the SMART study, the five most commonly isolated Gram-negative pathogens from IAIs were *E. coli*, *K. pneumoniae*, *P. aeruginosa*. *Enterobacter cloacae* and *P. mirabilis* (Table 1). This was very similar across all geographical regions, although *Acinetobacter baumannii* was more prevalent than *P. mirabilis* in Asia (4.6% *vs.* 2.1%) and *K. oxytoca* more prevalent than *P. mirabilis* in the South Pacific (3.8% *vs.* 3.0%, data not shown). The same species were also the most frequent pathogens associated with UTIs (Table 2) with no difference between geographical regions.

**Table 1:** Top five most prevalent Gram-negative bacteria associated with intra-abdominal infections from SMART between 2002 and 2011.

IAI Pathogen	N	%
*Escherichia coli*	43,973	47.8
*Klebsiella pneumoniae*	13,385	14.5
*Pseudomonas aeruginosa*	8,674	9.4
*Enterobacter cloacae*	5,564	6.0
*Proteus mirabilis*	3,282	3.6
Other	17,208	18.7
Total	92,086	100.0

**Table 2:** Top five most prevalent Gram-negative bacteria associated with urinary-tract infections from SMART between 2009 and 2011.

UTI Pathogen	N	%
*Escherichia coli*	10,956	44.3
*Klebsiella pneumoniae*	2,906	11.8
*Pseudomonas aeruginosa*	1,331	5.4
*Proteus mirabilis*	1,137	4.6
*Enterobacter cloacae*	617	2.5
Other	7,758	31.4
Total	24,705	100.0

**Figure 2:**
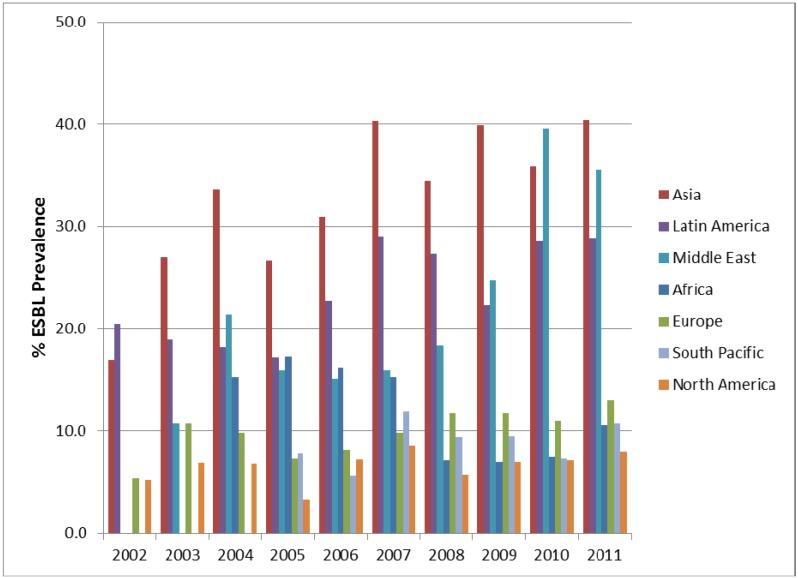
Prevalence of ESBLs in *E. coli*, *K. pneumonia*, *K. oxytoca* and *P. mirabilis* from intra-abdominal infections by region from SMART 2002 to 2011.

**Figure 3:**
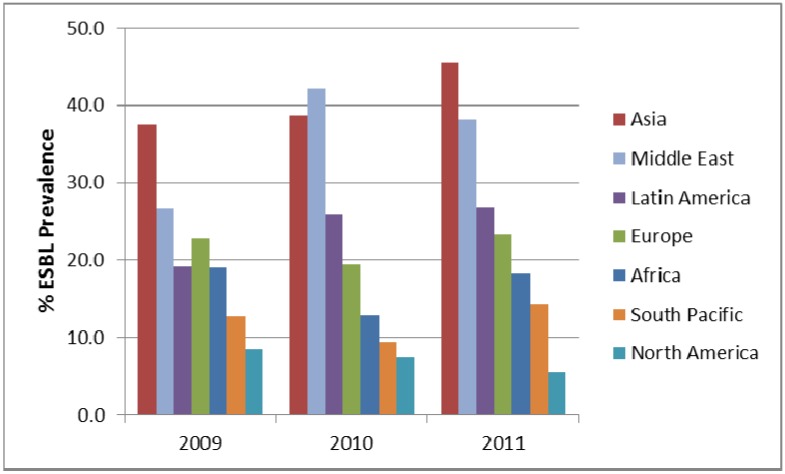
Prevalence of ESBLs in *E. coli*, *K. pneumonia*, *K. oxytoca* and *P. mirabilis* from urinary-tract infections by region from SMART 2009 to 2011.

One particular cause for concern is the prevalence of ESBLs in the clinical setting. In the SMART study, all *E. coli*, *K. pneumoniae*, *K. oxytoca* and *P. mirabilis* were phenotypically characterised for the presence of ESBL and carbapenemase enzymes. The prevalence of ESBLs in IAIs in the SMART study by region is shown in Figure 2 and Figure 3. It can be seen that ESBL rates are particularly high in Asia, Latin America and the Middle East, which has been noted elsewhere [5]. The prevalence of ESBLs intra-abdominal infections has steadily increased over time for in Asia. Europe, Latin America, Middle East (all *p*-values < 0.0001), North America (*p*-value = 0.03) and South Pacific (*p*-value = 0.009). Interestingly the trend for ESBls in intra-abdominal infection isolates from Africa is a decreased over time, which is statistically significant (*p*-value = 0.037). For UTI pathogens the changing prevalence of ESBLs is less dramatic with only significant increases found in the Middle East (*p*-value < 0.0001) and Asia (*p*-value = 0.0002).

Molecular analysis of ESBLs and also carbapenemases is an important component of the SMART study and details of the methodology employed are described by Lascols *et al.* [6]. A recent analysis of ertapenem-resistant isolates from SMART indicate that a range of carbapenemase genes, associated with numerous ESBLs and/or AmpC backgrounds, were found among *Enterobacteriaceae* isolated during the study. Many of these ertapenem non-susceptible strains were clonally related and carried various combinations of β-lactamases [7]. The most common carbapenemase found in isolates collected in Indian hospitals was NDM-1 [7], despite the fact that this carbapenemase was only discovered fairly recently [8]. This carbapenemase was found on a diverse collection of *Enterobacteriaceae* [7], as describe by others previously [9,10,11]. A separate analysis of isolates from the Asia-Pacific region found that carbapenemases were almost exclusively detected among the *Enterobacteriaceae* isolates collected from India, with some IMP β-lactamases also detected in the Philippines and Australia [12]. In this study, *K. pneumoniae* had a slightly lower higher resistance rate to imipenem compared to *E. coli* (Figure 6, Figure 7, Figure 8 and Figure 9). The main carbapenem resistance mechanisms involve a KPC enzyme, usually in combination with an SHV [7]. Surveillance studies, such as the SMART program, process a large number of bacteria providing the opportunity to identify and track novel resistance mechanisms. Our extensive molecular analysis has identified a new *bla*_SHV_ variant, *bla*_SHV-129_, and a new *bla*_KPC_ variant, *bla*_KPC-11_, as well as confirming the dominance of certain ESBL types, *i.e.*, CTX-M-15 [6]. Surveillance studies are important in detecting changes in resistance and epidemiology as well as the future evolution of bacterial resistance.

**Figure 4:**
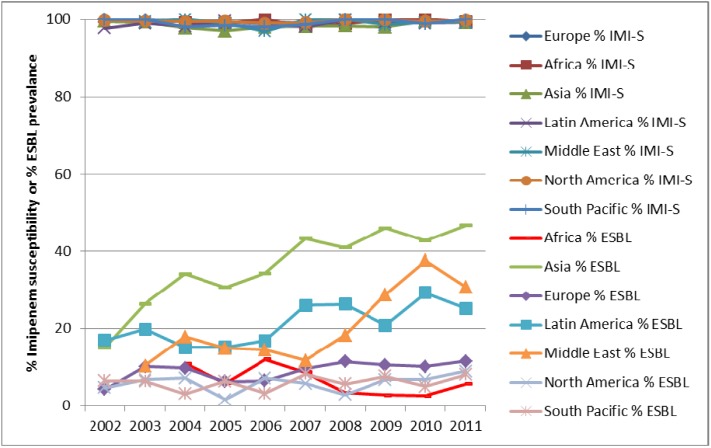
Susceptibility of *E. coli* from intra-abdominal infections to imipenem (IMI-S) and prevalence of ESBLs by region from SMART 2002 to 2011.

**Figure 5:**
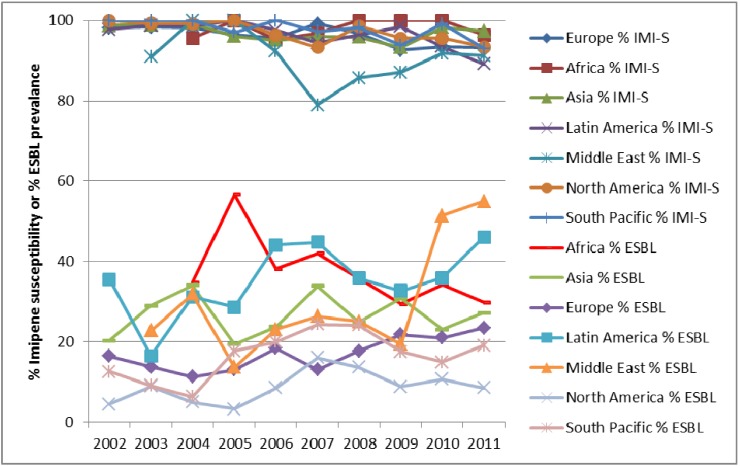
Susceptibility of *K. pneumoniae* from intra-abdominal infections to imipenem (IMI-S) and prevalence of ESBLs by region from SMART 2002 to 2011.

**Figure 6:**
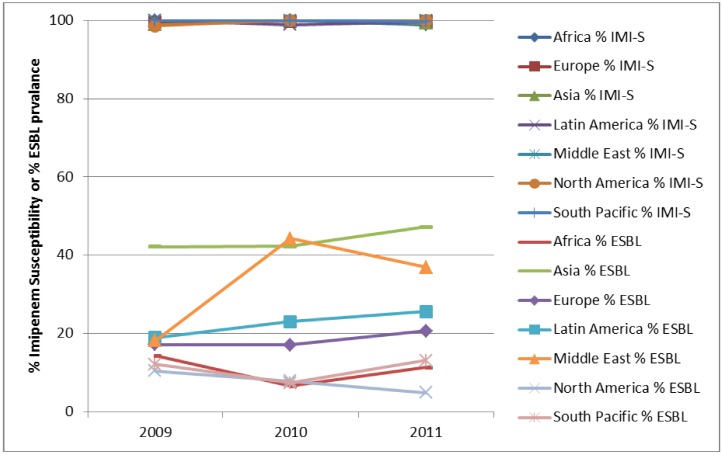
Susceptibility of *E. coli* from urinary-tract infections to imipenem (IMI-S) and prevalence of ESBLs by region from SMART 2009 to 2011.

*E. coli* isolates from both IAIs and UTIs demonstrate consistently low resistance to carbapenems (as demonstrated by imipenem in Figure 4, Figure 5 and Figure 6) since the beginning of SMART. Other antimicrobial agents are less active, especially against ESBL-positive *E. coli*.

In addition to the expected increased resistance to β-lactams, fluoroquinolone resistance in ESBL-positive *E. coli* causing intra-abdominal infections ranges from 60% to 93% in India, China, North America, Europe and South Africa [7,13,14,15,16,17,18]. Furthermore, the vast majority of Gram-negative pathogens causing UTIs are resistant to fluoroquinolones [19,20]. For all antimicrobial agents, apart from carbapenems, it has been observed that *E. coli* from hospital-associated infections are less susceptible compared to community-associated infections [21].

**Figure 7:**
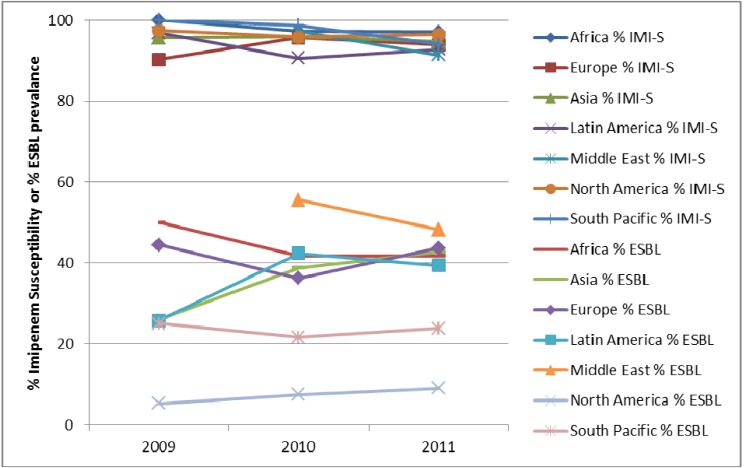
Susceptibility of *K. pneumoniae* from urinary-tract infections to imipenem (IMI-S) and prevalence of ESBLs by region from SMART 2009 to 2011.

*K. pneumoniae* also continue to remain susceptibile to carbapenems, especially those from UTIs (Figure 7). Although carbapenem activity against isolates from IAIs is also high (Figure 5), it is slightly lower than activity against *K. pneumoniae* isolates from UTIs or *E. coli*. Carbapenem activity was highest among the tested antimicrobial agents as recently demonstrated against *K. pneumoniae* from China where cephalosporins, ampicillin-sulbactam, and fluoroquinolones have 40%–50% resistance [13], presumably related to the high ESBL rates in this region. In other parts of the world, ESBL-positive *K. pneumoniae* are highly resistant to fluoroquinolones and β-lactams [14,15,16,21], as described with *E. coli.* Also, as with *E. coli*, *K. pneumoniae* causing hospital infections are more resistant to antimicrobial agents than those that are community-associated [21].

**Figure 8:**
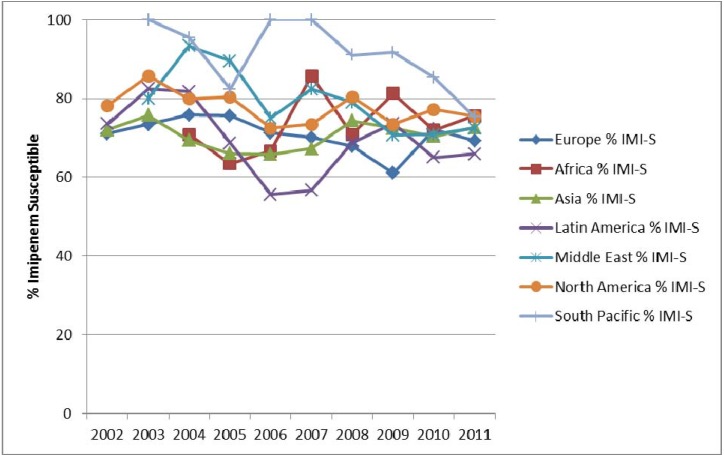
Susceptibility of *P. aeruginosa* from intra-abdominal infections to imipenem (IMI-S) by region from SMART 2002 to 2011.

**Figure 9:**
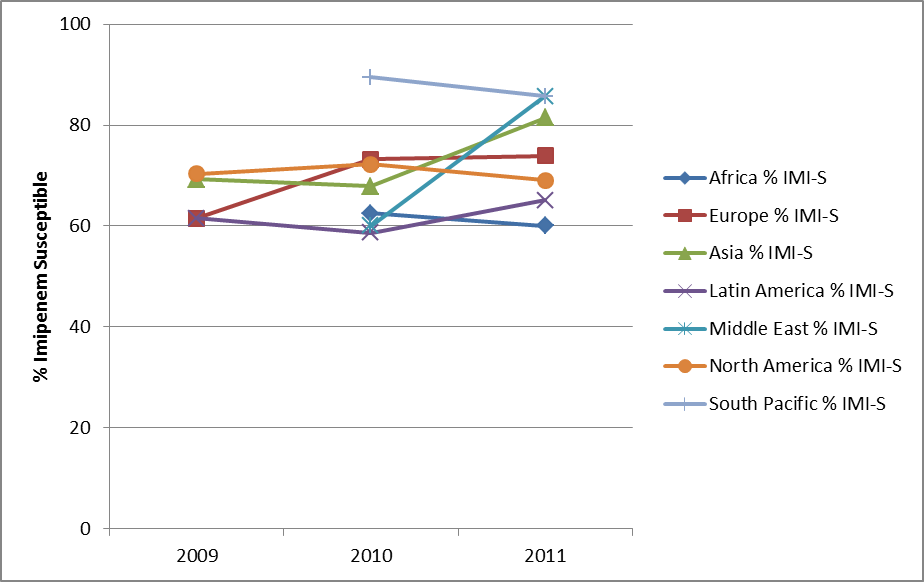
Susceptibility of *P. aeruginosa* from urinary-tract infections to imipenem (IMI-S) by region from SMART 2009 to 2011.

*P. aeruginosa* was the third most common pathogen with IAIs and UTIs at a rate of 5 and 10%, respectively (Table 1 and Table 2). Imipenem resistance ranged between 20% and 40% in these infection types in various regions of the world (Figure 8 and Figure 9). Although overall lower carbapenem resistance was observed among *E. coli* and *K. pneumoniae* compared with *P. aeruginosa*, the activity of imipenem was greater against *P. aeruginosa* compared to most other classes of antimicrobial agents. The resistance of *P. aeruginosa* to fluoroquinolones has significantly increased over time in North America from approximately 22% in 2005 to 33% in 2010 while imipenem activity remained unchanged at 20% resistance. During this ten-year period, resistance to piperacillin-tazobactam, cefepime and ceftazidime remained unchanged at 23 to 26% [22].

Interestingly, antibiotic resistance decreased over time for amikacin where resistance decreased from 10.6% in 2005 to 3.3% in 2010 in North America [22]. The relative activity of antimicrobials is not consistent between different regions of the world. For example, in South Africa, piperacillin-tazobactam resistance in *P. aeruginosa* was 8% but cefepime, ceftazidime and imipenem resistance was around 25 to 26%. Furthermore, in contrast to the much higher rate in North America, amikacin resistance in this species in South Africa was 27% [16]. In China (2009), the resistance of *P. aeruginosa* to amikacin was 12% whereas piperacillin-tazobactam was 8% [13]. Overall, all antimicrobial classes were less effective against *P. aeruginosa* hospital-associated infections compared to community-associated infections [21].

Clinical microbiologists and surveillance studies rely on accurate breakpoints to determine resistance rates of bacteria to antimicrobial agents based upon pharmacological and epidemiological data. These breakpoints are subject to change as new clinical or pharmacokinetic data are provided to the scientific community. CLSI and EUCAST update breakpoints annually. In 2010 CLSI reduced the breakpoints for carbapenems against *Enterobacteriaceae*, such that the susceptible breakpoint for ertapenem was reduced from 2 to 0.25 μg/mL [23]. Therefore, isolates with ertapenem MIC of 0.5 and 1 μg/mL were no longer considered susceptible. However, subsequent clinical data and MIC distributions and molecular analysis from the SMART study indicated that isolates with ertapenem MIC of 0.5 μg/mL do not contain carbapenemases and therefore should respond to ertapenem therapy [24]. Based upon these data, the CLSI susceptible breakpoints for ertapenem were increased to 0.5 µg/mL in 2012 [25]. Data derived from the SMART study provided essential information for determining the most appropriate epidemiological breakpoints for clinical use.

## 4. Conclusions

The SMART study has become an invaluable resource to track the resistance of Gram-negative bacteria to antimicrobial agents over time and a contemporary view of the most relative prevalence of the pathogens associated with IAIs and UTIs. Importantly, the assessment of molecular mechanisms of resistance is vital not just for epidemiological purposes but also for determining appropriate breakpoints of antimicrobial agents critical to patient care. The data produced have been able to highlight the differences in antimicrobial agent resistance between various regions of the world and these data demonstrate the continued utility of carbapenems in the treatment of these infections.
